# Opposing effects of prior information on relational representation and visual cues in dynamic social interaction perception

**DOI:** 10.1177/20416695251340298

**Published:** 2025-05-11

**Authors:** Yun Chen, Xin-Yu Xie

**Affiliations:** 12655 East China Normal University, Shanghai, China

**Keywords:** social interaction, relational representation, serial dependence, adaptation, priors

## Abstract

Social interaction, as a crucial component of relational representation, is essential for understanding human social cognition. While visual cues play a pivotal role in perceiving interactions, little is known about how individuals utilize past visual and interaction-related relational judgments when making decisions under uncertainty. This study investigated how past visual information and interpersonal relational judgments influence the current interaction perception. Participants continuously evaluated the interaction state of two avatars presented at varying distances and facing orientations. The findings revealed a dissociation where the perception of the current interaction state tends to be biased toward past interaction states rather than past distance cues, and this only occurs when the prior interaction information comes from the same sensory modality and is consciously attended to. For the distance cues that contribute to interaction representation, the current distance perception deviates from past distance, even when distance was not explicitly processed. This opposite influence of past information on visual cues and interaction relational representation reflects two independent processing mechanisms of prior information. When dynamically perceiving interpersonal interactions, individuals integrate the repulsive effect of visual cues with the attractive effect of past interaction relations to form stable interaction perception.

## How to cite this article

Chen, Y., & Xie, X-Y. (2025). Opposing effects of prior information on relational representation and visual cues in dynamic social interaction perception. *i-Perception, 16*(0), 1–15. https://doi.org/10.1177/20416695251340298

## Introduction

Human perception involves not only recognizing individual objects but also perceiving relations between them (Hafri & Firestone, 2021). Identifying social interactions, as an essential type of relational representation (Hafri & Firestone, 2021; Malik & Isik, 2023), is particularly crucial for navigating complex social environments. Previous studies have highlighted that visual cues, such as interpersonal distance and facing orientation, strongly influence judgments of social interaction (Heider & Simmel, 1944), with greater distances and less direct orientations typically reducing perceived interactivity (Zhou et al., 2019). Human visual system can rapidly detect interactions (McMahon & Isik, 2023), and like other forms of relational perception, this process involves both the representation of specific visual features and the relational structures formed from them.

However, prior research has largely concentrated on static or isolated judgments of interaction states, either via static images (Arioli et al., 2021; Quadflieg & Koldewyn, 2017) or brief video segments (Isik et al., 2017; Lee Masson & Isik, 2021). Real-world social interactions, by contrast, are inherently dynamic and require observers to continuously integrate incoming visual information with prior relational representations. To accurately perceive ongoing interactions, individuals must constantly update their perception based on prior experiences, balancing stability with responsiveness to new information (Frith, 2007; Hari et al., 2015). Consequently, the critical question remains: how does prior relational information shape current interaction perception, and how does the brain balance stability against sensitivity to new, incoming visual cues?

Recent research highlights two distinct mechanisms through which prior experiences influence current perceptual judgments: serial dependence and adaptation. Serial dependence refers to an attractive perceptual bias, helping individuals maintain stability by integrating recent experiences into current judgment (Cicchini et al., 2014; Fischer & Whitney, 2014). This attractive bias has been widely observed across various sensory and social domains and supports perceptual continuity in a stable world (for reviews, see Cicchini et al., 2024; Pascucci et al., 2023). In contrast, adaptation introduces a repulsive bias, enabling sensitivity to changes by reducing neural responses to repeated or constant stimuli, thus facilitating the detection of subtle changes (Gibson & Radner, 1937; Webster, 1996; Webster & Mollon, 1991).

These effects serve complementary perceptual functions: serial dependence optimizes perception by leveraging past regularities (Cicchini et al., 2018), while adaptation enhances responsiveness to novel stimuli (Webster, 2011). Existing research suggests that both attractive serial dependance effects and repulsive adaptation effects may coexist (Fritsche et al., 2017, 2020). However, it remains debated under which specific experimental conditions prior information exerts an attractive or repulsive effect on current perceptual processing. For example, Fritsche et al. (2020) found that recent priors tend to exhibit an attractive effect, while longer-term prior shows a repulsive effect. However, other studies have reported the opposite results (Chopin & Mamassian, 2012). Additionally, research has shown that for the same facial stimulus, gender processing exhibits an attractive effect, whereas expression processing shows a repulsive effect (Taubert et al., 2016), suggesting that perception of stable features remains consistent, while perception of transient features is more sensitive to changes in those features.

These findings point to different encoding and decoding mechanisms in the brain for prior information (Fritsche et al., 2020). Positive serial dependence effects often represent the perceptual decision-making process (Ceylan et al., 2021; Fritsche et al., 2017), but they also influence the early processing stages of current stimuli (Cicchini et al., 2021) and may be tied to the sensory modality through which the stimuli are presented (Fornaciai & Park, 2019). This suggests that serial dependence is not solely a postperceptual decision process but also interacts with early sensory input processing. This phenomenon may represent an optimal strategy based on Bayesian inference, where past events serve as good predictions of the future in contexts where environmental stability is assumed, as humans often rely on the statistical regularities of their immediate surroundings to interpret sensory input (Burr & Cicchini, 2014; Van der Burg et al., 2019). In contrast, visual adaptation primarily occurs at early stages of information processing (Blake et al., 2006) and is mainly due to long-term changes in neural response gain to specific attributes (Solomon et al., 2004). Negative aftereffects can be beneficial for amplifying changes, enabling the quick and sensitive detection of sensory updates.

In perceiving interpersonal interaction relations, these two mechanisms—serial dependence and adaptation—may operate differently depending on the type of prior information and task demands. Stable relational representations, such as social interaction states, typically evoke serial dependance effects, promoting perceptual continuity (Manassi & Whitney, 2024). Conversely, rapidly changing visual features, such as interpersonal distance or orientation, tend to induce adaptation effects, enhancing sensitivity to subtle changes. Social interaction perception can also involve cues from multiple sensory modalities (Campanella & Belin, 2007), raising an important question: do prior interaction representations formed through auditory cues influence subsequent interaction judgments based on visual input? Investigating this issue will clarify whether relational representations underlying interaction perception are modality-specific or modality-independent. Currently, however, it remains unclear how serial dependence and adaptation mechanisms jointly shape dynamic interpersonal interaction perception.

In this study, we designed a third-person perspective interaction judgment task to investigate how prior interaction judgments and visual cues influence current interaction perception. Specifically, we aimed to determine whether perception of current interactions is biased toward previous interaction states (serial dependence) or repelled away from them (adaptation). Additionally, we sought to distinguish whether such biases originate from prior relational representations themselves or merely reflect adaptation to the visual cues, such as distance cues, comprising these judgments. Further, we explored whether prior interaction representations formed in one sensory modality (e.g., auditory) affect interaction judgments in another modality (e.g., visual). By addressing these questions, we aimed to reveal how individuals dynamically integrate prior relational representations and sensory cues to maintain stable and accurate perception of interpersonal interactions, especially under conditions of ambiguous or uncertain visual information.

## Method

### Participants

The experiment included four tasks, each using a 2 × 2 within-subjects design (two types of prior stimuli, one-back or two-back). We aimed to investigate whether different prior stimuli in one-back or two-back conditions affect current perception. Previous research typically employed a medium effect size in similar prior effects (Fritsche et al., 2020; Taubert et al., 2016), so we selected a medium effect size of f = 0.25, with α = .05 and power = 0.80. G*Power calculations indicated a minimum of 24 participants per task. Considering the exclusion of invalid data, we aimed for at least 30 participants per task. The final sample sizes were 40 participants (22.2 ± 1.8 years old, 15 males, 25 females) for Task 1, 40 participants (22.2 ± 1.8 years old, 15 males, 25 females) for Task 2, 37 participants (22.2 ± 1.8 years old, 12 males, 25 females) for Task 3, and 30 participants (22.4 ± 1.7 years old, 13 males, 17 females) for Task 4. All participants were right-handed with normal or corrected-to-normal vision and normal audition. All were naive to the purpose of the tasks. Participants were paid and provided written informed consent before starting the experiment. The study has been approved by the University Committee on Human Research Protection at East China Normal University.

### Apparatus and Stimuli

Experimental measures were performed in a quiet room. Visual stimuli were generated using PsychoPy (Peirce, 2007) and presented on a 23.8-in. LCD monitor (Redmi RMMNT238NFS) at a resolution of 1600 × 900 pixels and a refresh rate of 60 Hz. Auditory stimuli were generated using Pydub and delivered by external speakers (EDIFIER R12U). Participants sat 1 m from the screen.

**Figure 1.**
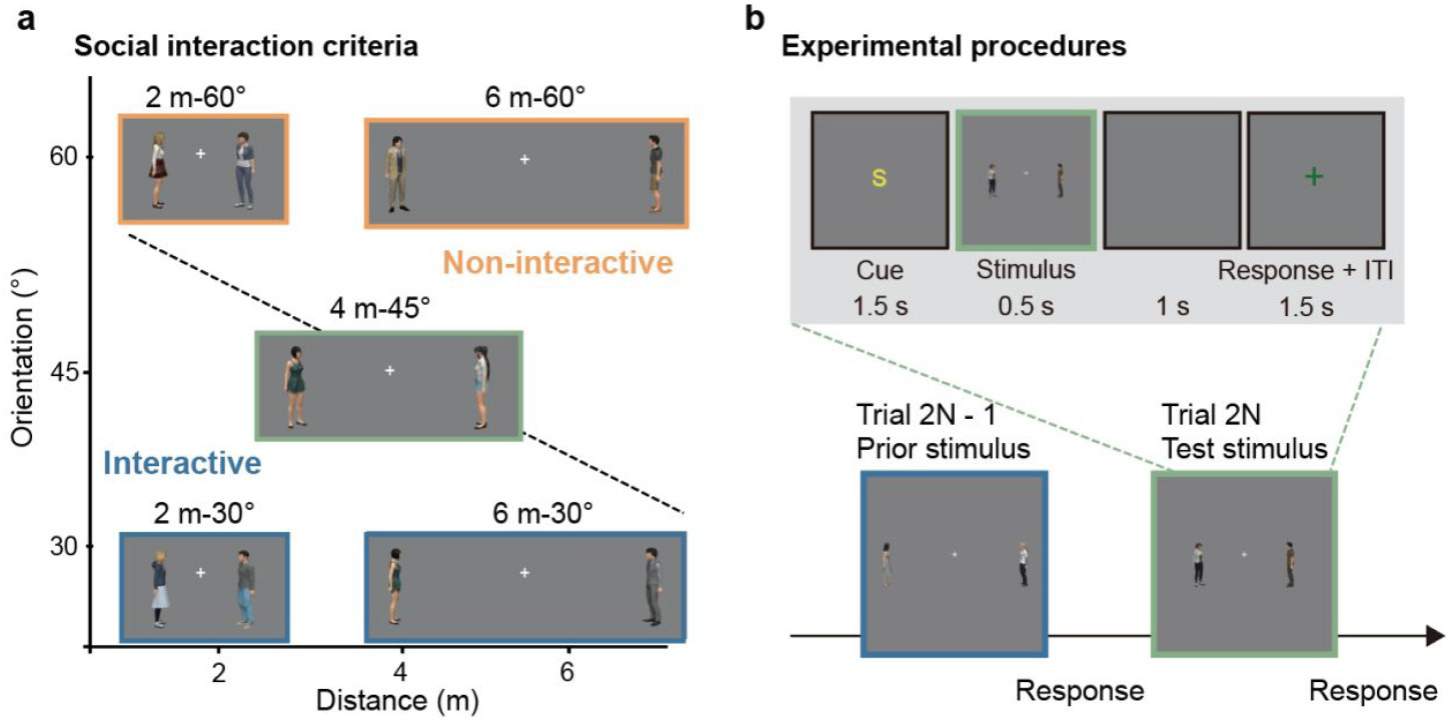
Stimuli and task paradigm. (a) Examples of avatar pairs with different distance-orientation. We defined the social interaction criteria as the black dashed line, so the avatar pairs below it are labeled as interactive (blue frame), and those above it are labeled as noninteractive (orange frame). (b) Task paradigm. In each task, prior trials and test trials alternated. A cue was presented before the prior or test stimulus: cue “S” indicated a visual social interaction task, cue “D” indicated a distance task, and cue “A” indicated an auditory social interaction task. For visual social interaction or distance tasks, the prior stimulus was a 2 m-75° or 6 m-15° avatar pair. In the auditory social interaction task, the prior stimulus was an interactive or noninteractive speech segment. The test stimulus was always 4 m-45° avatar pair across all tasks.

The visual stimulus was a pair of avatars. A total of 25 male or female avatars were generated utilizing the morph character system within Unity 3D (Unity Technologies). The avatars’ heights were set at approximately 1.75 m, and they were situated within a standard gray background. Two avatars stood opposite each other with their hands naturally down. The facing direction of an avatar could be 0°, 15°, 30°, 45°, 60°, 75° (0 indicates facing left or right). In each trial, two avatars were randomly selected to form an avatar pair, symmetrically positioned on the screen left and right, with varying distances. One avatar was fixed at 0°, while the other was rotated at a specific angle to create different orientations (Figure 1a).

The auditory stimulus was a 3-s interactive or noninteractive audio segment of a single person speaking. For example, an interactive speech was like “It's been a long time since I've played sports, let's go play badminton together on Saturday.” or “I like swimming and jogging, what sports do you like?,” while a noninteractive speech was like “A black hole is a mysterious and captivating object in the universe” or “How can we ensure the reliability of AI in medical diagnosis?.” There were 30 segments for each interactive and noninteractive speech, each consisting of 17–20 Chinese characters, with the questions and statements evenly distributed. Each audio segment included male and female versions, generated using AI dubbing with consistent emotions. One segment was randomly chosen in each trial. Across the task, all 60 segments appeared once, with an equal split between male and female voices.

### Procedures

To provide parameter selection guidance for the subsequent experiment, we conducted a preliminary experiment. Six participants, different from those in the formal experiment, were tasked with judging interaction perception for avatar pairs with varying distances and orientations (24 combinations in total, see Supplemental Materials for details). Based on the results of this preliminary experiment (Supplemental Figure S1), 2 m-75° avatar pair was selected as the clear noninteractive prior stimulus, and 6 m-15° as the clear interactive prior stimulus. The main experiment consisted of training and testing phases.

#### Training Phase

Before the formal testing, there was a training phase to help participants reinforce the appropriate interactive judgement criteria. During the training phase, participants were presented with eight pairs of avatars with varying distance-orientations: 2 m-30°, 4 m-30°, 6 m-30°, 2 m-45°, 6 m-45°, 2 m-60°, 4 m-60°, and 6 m-60°. We categorized these stimuli into two interactive states: the first four pairs were interactive, and the last four were noninteractive (see the criteria in Figure 1a). On each trial, after a cue “S” was presented for 1.5 s, the stimulus was presented for 0.5 s, followed by a blank screen for 1 s. When a green fixation cross appeared, participants needed to press the left or right arrow to judge whether the avatar pair was interacting. A “correct/incorrect” feedback was given for 0.4 s after the response.

The training phase consisted of two blocks, with each block containing 64 trials. Eight types of avatar pairs were randomly presented within each block (eight trials for each pair) to ensure that participants maintained stable judgement criteria.

#### Testing Phase

During the testing phase, a prior stimulus and a test stimulus were alternately presented (Figure 1b). The prior stimulus was a 2 m-75° or 6 m-15° avatar pair (which did not appear during training and was easier to judge compared to the training stimuli) or interactive/noninteractive speech, and the test stimulus was always a 4 m-45° avatar pair. There were four combinations of prior and test task sets: visual social interaction to visual social interaction (VSI-VSI, Task 1), distance to VSI (DIST-VSI, Task 2), audio social interaction to VSI (ASI-VSI, Task 3), and VSI to DIST (VSI-DIST, Task 4) (see Table 1). Each participant completed at least one task. The general procedure was similar across all tasks.

**Table 1.** Experimental Conditions.

Experiment	Prior Task	Prior Stimulus		Test Task	Test Stimulus
		Press Yes	Press No		
Task 1: VSI-VSI	Visual social interaction	6 m-15°	2 m-75°	Visual social interaction	4 m-45°
Task 2: DIST-VSI	Distance judgment	6 m-15°	2 m-75°		
Task 3: ASI-VSI	Auditory social interaction	Interactive speech	Noninteractive speech		
Task 4: VSI-DIST	Visual social interaction	6 m-15°	2 m-75°	Distance judgment	4 m-45°

*Note.* VSI-VSI = visual social interaction to visual social interaction; DIST-VSI = distance to visual social interaction; ASI-VSI = audio social interaction to visual social interaction; VSI-DIST = visual social interaction to distance.

In the VSI-VSI task, a cue “S” was presented for 1.5 s at the beginning to inform participants to judge whether the avatar pair was interacting. Then a 0.5 s prior stimulus (2 m-75°/6 m-15°) or test stimulus (4 m-45°) was presented, followed by a 1 s blank screen. When a green fixation cross appeared, participants pressed the left (No) or right (Yes) arrow key to judge the interactive state of the avatars. Participants were required to respond within 1.5 s, and no feedback was provided after each trial (Figure 1b). For participants, there were no “correct” or “incorrect” responses, nor was there a distinction between prior and test stimuli.

In the DIST-VSI task, a cue “D” was presented at the beginning of each prior trial to indicate that participants should judge whether the distance between two avatars exceeded the standard distance of 4 m. The standard distance was shown for 3 s at the beginning of each block. The prior stimuli were identical to those in the VSI-VSI task. Participants pressed the left (No) or right (Yes) arrow key to judge the distance of the avatars. This design ensured consistent key presses across both the VSI-VSI and DIST-VSI tasks, with participants pressing the right arrow key for 6 m-15° and the left arrow key for 2 m-75°. The test stimuli and task were identical to those in the VSI-VSI task.

In the ASI-VSI task, a cue “A” was presented at the start of each prior trial, instructing participants to judge whether the auditory speech content occurred during a two-person daily interaction or not. The prior stimulus was interactive/noninteractive speech, while the test stimulus remained the 4 m-45° avatar pairs used in the VSI-VSI task.

In the VSI-DIST task, the prior task required participants to judge VSI, while the test task required them to judge distance. The prior stimulus consisted of 2 m-75° or 6 m-15° avatar pairs, and the test stimulus was the 4 m-45° avatar pairs.

Each task consisted of two blocks, with each block containing 60 trials. The two types of prior stimuli, 2 m-75°/6 m-15° or interactive/noninteractive speech, appeared randomly in each trial (15 trials for each). Participants completed a total of 120 trials for each task (30 test trials for each type of prior).

### Data Analysis

To ensure that participants’ perception of the prior stimulus was clear and unambiguous, those with accuracy rates below 70% on prior tasks were excluded. As a result, four participants were excluded from the VSI-VSI task (leaving 36 participants), three from the DIST-VSI task (leaving 37 participants), three from the ASI-VSI task (leaving 34 participants), and five from the VSI-DIST task (leaving 25 participants). The remaining sample sizes for all tasks met the minimum requirements for statistical power.

To examine how current interaction perception of test stimulus is biased by prior stimulus (one-back), all test trials were categorized based on participants’ responses to the prior stimuli. In the VSI-VSI and DIST-VSI tasks, trials were divided into those following a “No” response to the 2 m-75° prior and those following a “Yes” response to the 6 m-15° prior. Similarly, in the ASI-VSI task, trials were categorized based on responses to noninteractive (“No”) or interactive (“Yes”) speech. For each category, we calculated the percentage of trials where the test stimulus was judged as interactive. To measure how current interaction perception is biased by previous test stimulus (two-back), the current test trials were similarly divided into two categories based on the responses to the previous test stimulus (interactive/noninteractive), and the percentage of trials judged as interactive was calculated for each category. In VSI-DIST task, both one-back and two-back effects were calculated in a similar way, assessing how the current distance perception is biased by the prior or previous test stimulus.

Statistics was performed in JASP (12.1.0). Paired-sample t-tests and mixed ANOVAs were used to measure the one-back/two-back effects.

## Results

### Social Interactions Perception Is Attracted to Previous Interaction Representation

**Figure 2.**
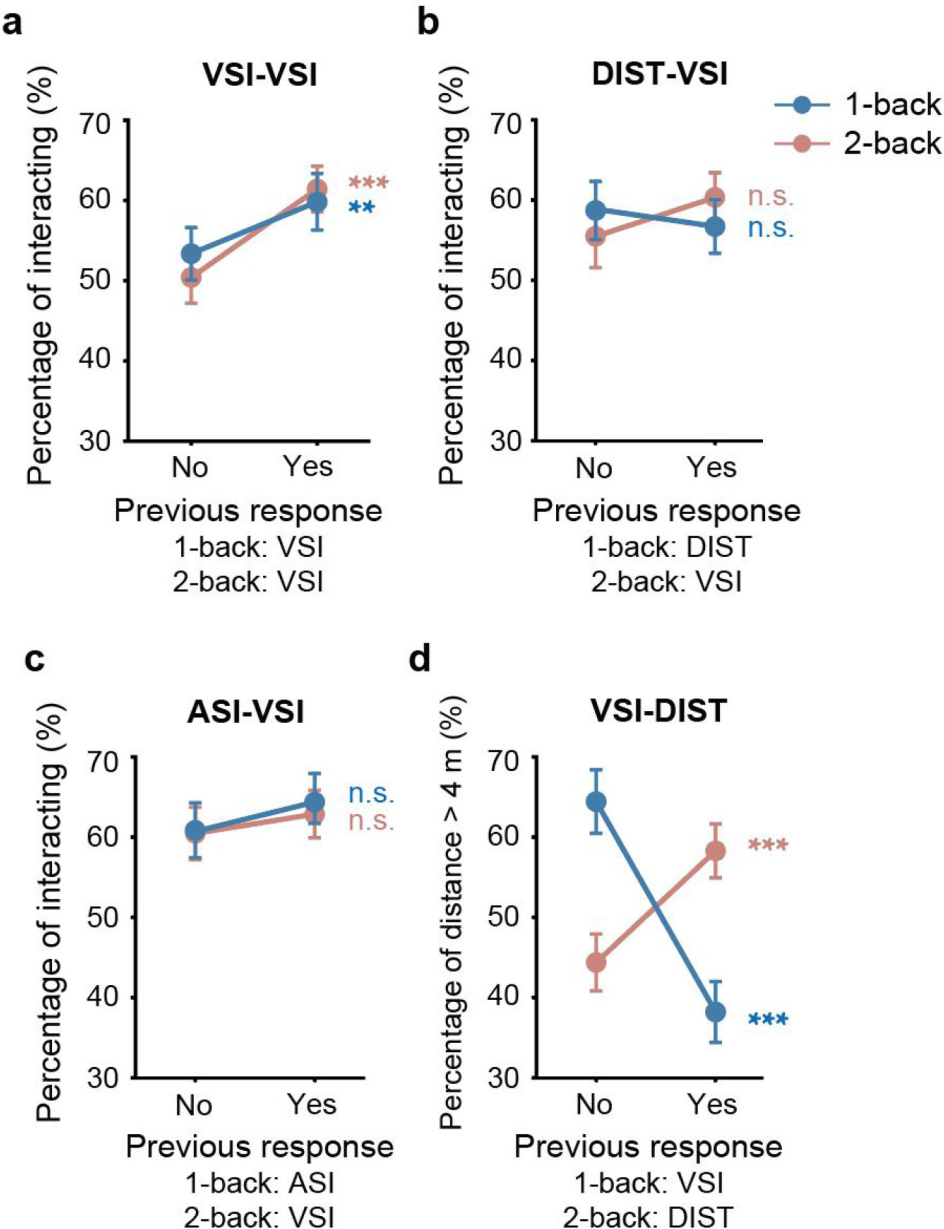
Experimental results. (a) VSI-VSI task: percentage of test stimuli judged as interactive, influenced by responses to the previous task (VSI in both one-back and two-back conditions). (b) DIST-VSI Task: percentage of test stimuli judged as interactive, influenced by responses to the previous task (DIST in one-back, VSI in two-back). (c) ASI-VSI Task: percentage of test stimuli judged as interactive, influenced by responses to the previous task (ASI in one-back, VSI in two-back). (d) VSI-DIST task: percentage of test stimuli judged as greater than 4 m, influenced by responses to the previous task (VSI in one-back, DIST in two-back). The “one-back” condition reflects the influence of responses to prior stimuli, while “two-back” reflects the influence of responses to the preceding test stimulus. Error bars represent the standard error of the mean across participants. **p < .05, ***p < .01.

*Note.* VSI-VSI = Visual Social Interaction to Visual Social Interaction; DIST-VSI = Distance to Visual Social Interaction; ASI-VSI = Audio Social Interaction to Visual Social Interaction; VSI-DIST = Visual Social Interaction to Distance.

This study investigated how individuals rely on prior information for social interaction judgments in the face of uncertainty. In the VSI-VSI task, participants were required to assess the interactive state of two avatars positioned at varying distances and orientations. A prior stimulus and a test stimulus were alternately presented during the task. For the prior stimulus, interaction information was explicit: only 3.2% of the 2 m-75° avatar pairs were judged as interactive, while 95.5% of the 6 m-15° pairs were considered interactive. The test stimulus, characterized by 4 m-45°, provided an ambiguous interaction signal, with a 56.6% probability of being judged as interactive. Crucially, participants relied on past interaction information when evaluating this ambiguous stimulus: if the prior involved a 6 m-15° interacted pair, as opposed to a 2 m-75° noninteraction prior, participants were more likely to report interaction (59.1% ± 3.4% vs. 53.3% ± 3.1%, *t* = 2.098, *p* = .043, Cohen's d = 0.350, Figure 2a, one-back).

Through experimental design, the observed attractive serial dependence effect rules out the possibility of being attributed to the attraction of prior distance cues. If the effect were distance-based, the 2 m-75° prior would have made the 4 m-45° test stimulus appear closer and more likely to be judged as interactive, which was not observed. In addition, the possibility of orientation attraction is low because the angular difference between the prior and test stimuli was 30°, and serial dependence for such orientation differences is typically weak (Cicchini et al., 2018; Fischer & Whitney, 2014).

To further clarify that the attractive serial effect indeed arises from interaction perception, we examined how participants’ judgments of the current test avatar pairs were influenced by their interaction judgments of the previous test avatar pairs. Results showed that when participants judged the previous test as interactive, they were significantly more likely to judge the current test as interactive compared to when they judged the previous test as noninteractive (60.9% ± 2.7% vs. 50.2% ± 3.0%, *t* = 4.807, *p* < .001, Cohen's d = 0.801, Figure 2a, two-back). Notably, the past and current test stimuli were identical (4 m-45°), indicating that the attractive effect from past information is related to the perception of past interaction relations rather than simply a bias towards previously presented distance or orientation cues.

### Explicitly Attended Interaction Relations Attract Social Interaction Perceptions From the Same Sensory Modality Input

It is currently unclear whether the attraction bias occurs as long as past stimuli contain interaction information or only occurs when past interaction relations are attended to. Therefore, participants were required to perform a DIST-VSI task, judging whether the distance in the prior stimulus (2 m-75° or 6 m-15°) was greater than 4 m, while also assessing the interactive state of the test stimulus (4 m-45°).

When participants focused their attention on distance in the prior task (with an accuracy rate of 98.0% ± 0.4%), their social interaction judgments of ambiguous test stimuli were unaffected by priors (56.2% ± 3.1% vs. 58.2% ± 3.4%, *t* = −0.888, *p* = .380, Cohen's d = −0.146, Figure 2b, one-back), despite the fact that the prior stimuli contained the same explicit interaction information as in the VSI-VSI task. However, we still observed some (although nonsignificant) attraction bias for social interaction in the two-back analysis (59.9% ± 2.9% vs. 55.0% ± 3.6%, *t* = 1.780, *p* = 0.084, Cohen's d = 0.293, Figure 2b, two-back), similar to the two-back effect in the VSI-VSI task, where the previous test stimulus remained a social interaction task.

We further sought to understand whether the attended interaction information in prior stimuli necessarily needs to be visual stimuli. Because previous research has demonstrated the absence of cross-modal serial dependence effects for simple features such as numerosity (Fornaciai & Park, 2019). However, it is unclear whether there are cross-modal effects for more abstract relationship representations like social interaction. To address this, we designed an ASI-VSI task, in which participants were required to determine whether the heard speech was from a daily interactive dialog or not (e.g., interactive speech: “I like swimming and jogging, what sports do you like?”; noninteractive speech: “A black hole is a mysterious and captivating object in the universe.”) in the prior task. In the subsequent test task, they still needed to judge whether the presented visual avatar pair (4 m-45°) indicated interaction.

The results revealed that while auditory stimuli contained significant interaction cues (with an accuracy rate of 95.6% ± 0.7%), they did not affect visual interaction perception. Participants’ judgments of the ambiguous test stimulus were not significantly biased toward the previous interactive speech (64.8% ± 3.1% vs. 60.8% ± 3.4%, *t* = 1.455, *p* = .155, Cohen's d = 0.250, Figure 2c, one-back), nor were the two-back effects significant (62.9% ± 3.0% vs. 60.5% ± 3.3%, *t* = 0.996, *p* = .326, Cohen's d = 0.171, Figure 2c, two-back). Despite both stimuli being visual in the two-back conditions, the interspersed auditory stimuli may still potentially disrupt the representation of visual interaction relations.

Results from the DIST-VSI and ASI-VSI tasks collectively indicate that only consciously attended interaction relations from the same sensory modality attracts current social interaction representation.

### The Representation of Visual Cues in Social Interaction Perception Is Repelled Away From Previous Visual Cues

Although previous results have shown that processing social interaction tends to be influenced by past interaction representations rather than visual cues, the possibility that the representation of visual cues themselves is also affected by prior stimuli cannot be ruled out. It may be that the subtle changes in visual cues are insufficient to significantly influence the interaction perception. To explore this, we employed a VSI-DIST task to examine whether interaction processing could bias the subsequent processing of distance cues.

The results revealed that, contrary to the attractive effect, when the prior stimulus contained an avatar pair at 6 m, participants were more likely to report the current 4-m stimulus as closer compared to when the prior was at 2 m (38.2% ± 3.8% vs. 64.4% ± 3.9%, *t* = −7.460, *p* < .001, Cohen's d = −1.492, Figure 2d, one-back), even when the prior task did not require direct distance judgment. This suggests that the processing of the current distance is significantly influenced by a repulsive effect of past distance information. However, in the two-back analysis, the representation of the current test distance significantly biased towards the distance of the previous test (58.3% ± 3.4% vs. 44.4% ± 3.6%, *t* = 4.871, *p* < .001, Cohen's d = 0.974, Figure 2d, two-back), suggesting that longer-term prior information exhibits an attractive effect in the processing of both current interaction and simple visual feature information.

**Figure 3.**
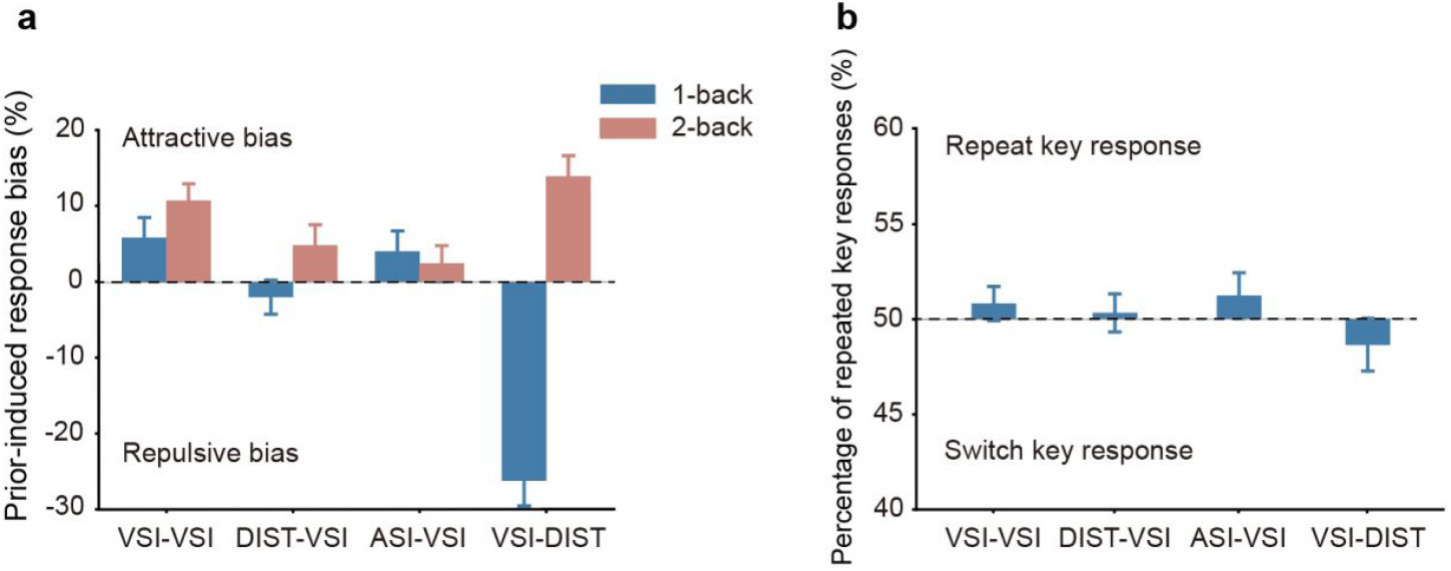
(a) Prior-induced response bias across experiments. The figure shows the attractive and repulsive biases for the four tasks (VSI-VSI, DIST-VSI, ASI-VSI, and VSI-DIST) under one-back (blue) and two-back (red) conditions. The attractive bias represents the perception shift toward the prior stimulus, while the repulsive bias represents the deviation from the prior stimulus. Error bars represent the standard errors of the mean across participants. (b) Percentage of repeated key responses across tasks. Error bars represent the standard errors of the mean across participants.

*Note.* VSI-VSI = Visual Social Interaction to Visual Social Interaction; DIST-VSI = Distance To Visual Social Interaction; ASI-VSI = Audio Social Interaction to Visual Social Interaction; VSI-DIST = Visual Social Interaction To Distance.

To further compare the effects across the four tasks, we calculated the prior-induced response bias for both the one-back and two-back conditions in each task. This bias was defined as the difference in the proportion of trials where participants judged the test stimulus as interactive (or distance greater than 4 m) when the previous response was “Yes” versus “No.” A bias greater than 0 indicates an attractive bias, where perception of the test stimulus is shifted toward the prior stimulus (Figure 3a). A mixed ANOVA was conducted with Previous Trial (one-back/two-back) as a within-subject factor and Task as a between-subject factor. The analysis revealed significant main effects for Previous Trial, F(1,128) = 43.976, *p* < .001, $\eta_{p}^{2}$ = 0.256, and Task, F(3,128) = 8.971, *p* < .001, $\;\eta_{p}^{2}$ = 0.174, as well as a significant interaction effect, F(3,128) = 20.478, *p* < .001, $\eta_{p}^{2}$ = 0.324. Post hoc analyses showed that, for the one-back condition, the bias in the VSI-DIST task was significantly different from the other tasks: *p*(VSI-DIST vs. VSI-VSI) < .001, *p*(VSI-DIST vs. DIST-VSI) < .001, *p*(VSI-DIST vs. ASI-VSI) < .001. However, for the two-back condition, there were no significant differences in bias between tasks: *p*(VSI-VSI vs. VSI-DIST) = 1.000, *p*(VSI-VSI vs. ASI-VSI) = .377, *p*(VSI-VSI vs. DIST-VSI) = 1.000.

Thus, only for the distance task, prior stimuli containing distance information leads to distance adaptation, even when the distance information was not explicitly processed. For the two-back condition, where both the past test and the current test involved the same task, an attractive bias was observed. Although the magnitude of the bias varied across tasks, this variation can likely be attributed to the interspersed tasks, which may have disrupted the retention of prior test information.

To ensure that the observed effects were not driven by a general tendency for participants to maintain or switch key responses, we examined key-press patterns from test to prior stimuli within each task. Since key responses from prior to test stimuli were the same as those analyzed in the serial dependance effect, only the key responses from test to prior were used to analyze participants’ motor response tendencies. The results showed no systematic bias toward response repetition or alternation. Specifically, participants exhibited no clear motor preference, as the proportion of trials in which they repeated versus switched key responses remained balanced (VSI-VSI: repeated proportion = 50.8% ± 0.9%, *p* = .343, compared to 50%; DIST-VSI: 50.3% ± 1.0%, *p* = .775; ASI-VSI: 51.2 ± 1.2%, *p* = .335; VSI-DIST: 48.6% ± 1.4%, *p* = .366; Figure 3b). These findings suggest that key-press tendencies did not systematically influence the observed effects.

## Discussion

When perceiving social interactions through visual cues, people integrate both current and past information. Past information can exert two opposing effects on social interaction perception: one involves treating previous interaction relations as prior knowledge, biasing current perceptions toward past interaction states, while the other involves visual cues adapting away from past visual cues to maintain sensitivity to changes in visual information. These distinct influences reflect how individuals balance perceptual stability and sensitivity to new information when making social interaction judgments.

Our findings suggest that attractive and repulsive effects may be governed by distinct mechanisms. The attractive effect is associated with higher-level processes (Cicchini et al., 2021; Fritsche et al., 2017), likely emerging after initial interaction representations are established. This is supported by findings that past interaction cues only significantly influence current judgments when participants explicitly evaluate interaction states, indicating that the attractive effect relies on cognitive processes beyond low-level perception (Bae & Luck, 2020). Additionally, the lack of cross-modal transfer in the attractive effect further indicates that prior interaction representations are modality-specific, rather than an abstract, modality-independent framework.

The attractive effect observed in our study does not result from an attraction toward low-level visual features such as distance, as our experimental design rules out this possibility. While we also hypothesize that orientation cues do not drive this effect—given previous research showing that serial dependence for orientation differences greater than 15° is typically weak (Cicchini et al., 2018; Fischer & Whitney, 2014)—it remains uncertain whether the 30° difference in relative facial orientation in our task completely eliminates the influence of prior orientation cues. However, the strong attractive effect in the two-back condition, where both prior and test stimuli were identical, provides compelling evidence that the effect is driven by past interaction representations rather than visual features. This reinforces the view that interaction perception is a structured, sensory-driven relational process rather than a simple accumulation of visual feature information.

The repulsive effect, however, largely originates from the early visual processing (Jin et al., 2005), as it occurs independently of task demands. This is evident in the VSI-DIST task, where a repulsive bias was observed, but not in the DIST-VSI task. This discrepancy suggests that distance, as a simple visual feature, can induce an automatic repulsive bias during early sensory processing. In contrast, interaction perception relies on task-relevant attention and active cognitive engagement, as social interaction information cannot be automatically extracted without deliberate cognitive processing.

Our results also indicate that the attractive effect of prior information may persist in working memory for an extended period (Chopin & Mamassian, 2012; Fischer & Whitney, 2014). Even after a two-back interval, prior information continued to significantly attract current perception. In contrast, the repulsive effect appears to be confined to short-term influences, reflecting how neuronal activity adapts to recent stimuli.

Although previous research has shown that the balance between attractive and repulsive effects may vary depending on stimulus reliability and uncertainty (Cicchini et al., 2018), the use of clear, noise-free stimuli in our study likely enhanced adaptation effects in distance judgments. However, the absence of adaptation in social interaction judgments suggests a fundamental difference between low-level sensory features and higher-level relational representations. Future studies can employ adaptation experimental paradigms to further investigate the mechanisms underlying interaction adaptation and the interplay between sensory and cognitive processes in dynamic social perception.

How the brain integrates the opposing influences of past interaction representations and visual cue representations in interaction perception remains a key challenge. Although previous research has shown that these two mechanisms coexist in neural activity (Hajonides et al., 2023; Sheehan & Serences, 2022; Zhang & Luo, 2023), studies using simple visual or auditory stimuli often struggle to disentangle their distinct contributions to brain representations. In contrast, the interactive stimuli used in our study may offer a clearer approach to distinguishing these processes. Given that early visual areas encode low-level sensory features while higher-order brain regions, such as the pSTS, represent interaction relations (Isik et al., 2017; Pitcher & Ungerleider, 2021), combining neuroimaging techniques with our paradigm could provide valuable insights into the mechanisms underlying dynamic perceptual processing in social interaction perception.

In addition, the avatars in the prior and test stimuli were different, yet we still observed robust attractive effects. This finding suggests that the attractive effect of prior interaction is not only independent of low-level visual features such as distance and orientation but may also be independent of the specific individuals depicted in the stimuli. However, we are uncertain whether such cross-individual transfer of attractive effects occurs in real-world social interaction perception. It is possible that real-world social interaction representations are inherently tied to the specific individuals involved. This difference might reflect a key distinction between the perception of virtual interactions and real-world interactions. Future research should explore this issue using more ecologically valid settings.

Dynamic recognition of social interactions is crucial not only for understanding human social behavior but also for developing humanoid robots. For service robots to feel friendly and responsive, they must accurately assess interactions among people, such as avoiding interruption in ongoing group interactions. While existing research allows robots to recognize static social interactions based on distance and orientation (Zhou et al., 2019, 2022), incorporating prior interaction information could enhance performance. For example, if two people are already interacting, a change in distance does not necessarily mean a change in their interaction state. Integrating prior information into robotic algorithms can improve computational efficiency and make robot responses more aligned with human perceptions, creating a more natural and effective interaction experience.

## Conclusions

This study reveals that in social interaction perception, prior information has opposing effects on interaction relation representations and visual cue representations. Specifically, interaction representations show an attractive effect, where current perceptions are biased toward past interactions states, whereas visual cues, such as distance, exhibit a repulsive effect, leading to deviations from past information. These contrasting influences suggest that different mechanisms govern the processing of relational representations and low-level visual features. Understanding how individuals integrate these conflicting effects is crucial for forming stable and accurate perceptions in dynamic social interactions. This study provides insights into the broader interplay between relational and sensory processing mechanisms in dynamic perception.

## Supplemental Material

sj-docx-1-ipe-10.1177_20416695251340298 - Supplemental material for Opposing effects of prior information on relational representation and visual cues in dynamic social interaction perception Supplemental material, sj-docx-1-ipe-10.1177_20416695251340298 for Opposing effects of prior information on relational representation and visual cues in dynamic social interaction perception by Yun Chen and Xin-Yu Xie in i-Perception
