# Supplementary material for: Opposing effects of prior information on relational representation and visual cues in dynamic social interaction perception
Source: Iperception. 2025 May 11;16(3):20416695251340298. doi: 10.1177/20416695251340298 (PMC12066850; doi:10.1177/20416695251340298)
Supplement: sj-docx-1-ipe-10.1177_20416695251340298 - Supplemental material for Opposing effects of prior information on relational representation and visual cues in dynamic social interaction perception [file sj-docx-1-ipe-10.1177_20416695251340298.docx]

# Supplementary Materials: Preliminary Experiment

To provide parameter selection guidance for the formal experiment, we conducted a preliminary study aimed at determining the appropriate distance and orientation combinations to use as prior stimuli in the main experiment. Six participants (different from those in the formal experiment) participated in this study. They were tasked with judging the interaction perception of avatar pairs presented at varying distances (1 m, 2 m, 4 m, 6 m) and orientations (0°, 15°, 30°, 45°, 60°, 75°), resulting in 24 unique combinations of distance and orientation. Each block included six repetitions of every combination, and there were six blocks in total, with the trial order randomized. Participants were instructed to decide whether the two avatars appeared to be interacting without any prior training or predefined judgment criteria.

The results of the preliminary experiment are summarized in Figure S1. As expected, both distance and orientation significantly influenced interaction judgments (Figure S1a). We further analyzed how participants' perception of the previous stimulus influenced their judgments of the current stimulus (Figure S1b). Across almost all stimulus combinations, participants were more likely to judge the current stimulus as interactive when the previous stimulus was perceived as interactive. This bias was particularly noticeable under conditions of high uncertainty.

However, due to the randomized presentation of stimuli in this preliminary experiment, it was difficult to distinguish whether this attractive effect stemmed from a bias toward past interaction representations or from the influence of prior distance or orientation processing on the current stimulus. To address this ambiguity in the formal experiment, we carefully selected specific stimuli that could minimize confounding factors and provide clearer insights into the mechanisms underlying prior effects.

For the formal experiment, the 4 m-45° combination was chosen as the test stimulus due to its high uncertainty, which is critical for evaluating prior effects. The 2 m-75° combination was selected as the clear non-interactive prior stimulus, as it consistently resulted in "No" responses (mean percentage of interaction = 28.7%) in the preliminary study. Similarly, the 6 m-15° combination was chosen as the clear interactive prior stimulus, as it consistently led to "Yes" responses (mean percentage of interaction = 75.9%, Figure S2). The large angular difference between the prior and test stimuli was designed to minimize potential orientation-based attraction effects, while the opposing effects of distance-based attraction and interaction representation attraction ensured that any observed biases would provide meaningful insights into how prior and current information is integrated.


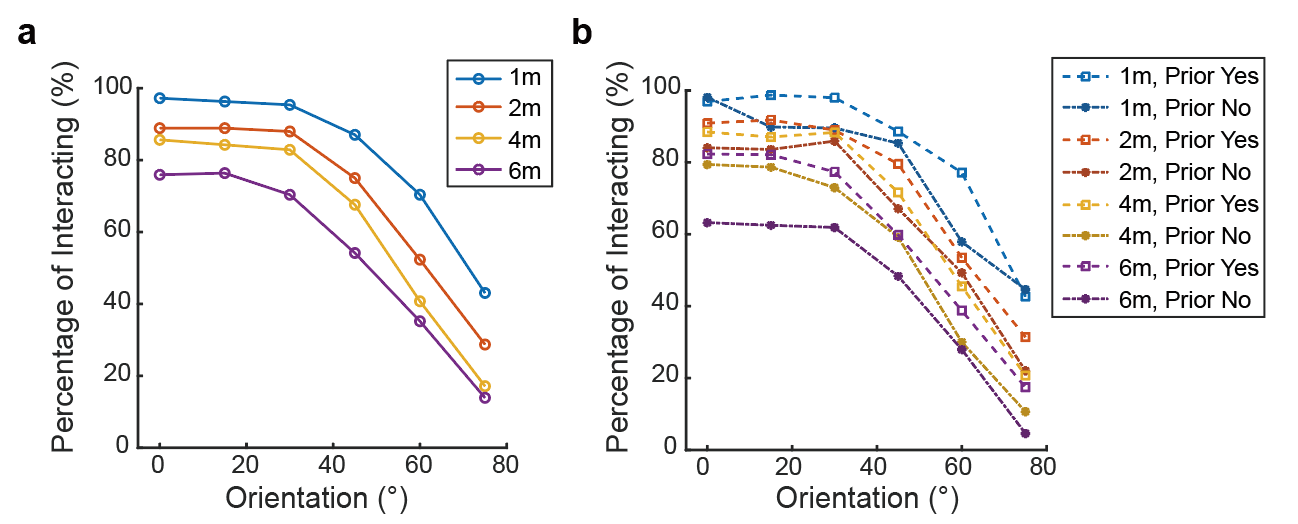


Figure S1. Results of Preliminary Experiment. (**a**) The percentage of trials judged as "interacting" across different combinations of distances (1 m, 2 m, 4 m, 6 m) and orientations (0°–75°). (**b**) The influence of prior interaction judgments ("Prior Yes" vs. "Prior No") on current interaction perception.


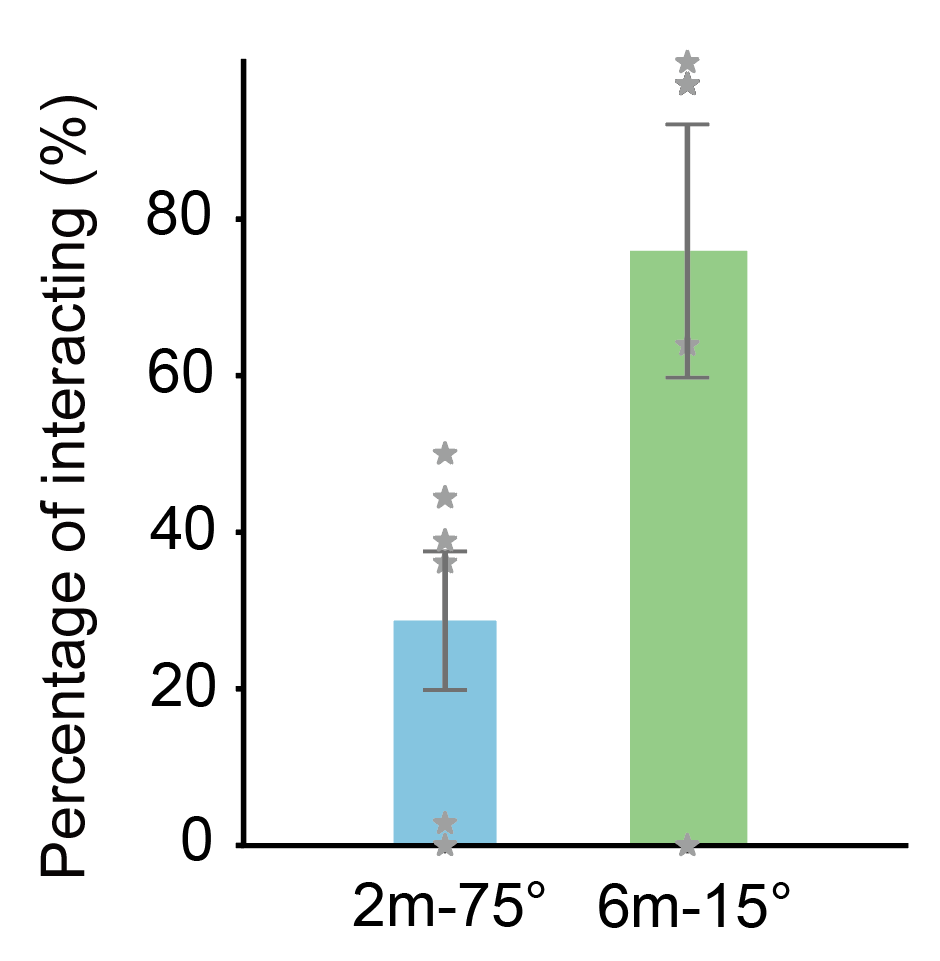


Figure S2. The mean percentage of interaction judgments for the 2m-75° and 6m-15° stimuli, which were selected as priors with clear interaction information in the formal experiment. Dots represent individual participant data, and error bars indicate standard error.
